# Genetic Association Analysis Using Sibship Data: A Multilevel Model Approach

**DOI:** 10.1371/journal.pone.0031134

**Published:** 2012-02-01

**Authors:** Yang Zhao, Hao Yu, Ying Zhu, Monica Ter-Minassian, Zhihang Peng, Hongbing Shen, Nancy Diao, Feng Chen

**Affiliations:** 1 Department of Epidemiology and Biostatistics, School of Public Health, Nanjing Medical University, Nanjing, Jiangsu, China; 2 Environmental and Occupational Medicine and Epidemiology Program, Department of Environmental Health, Harvard School of Public Health, Harvard University, Boston, Massachusetts, United States of America; 3 Imperial College Business School, Imperial College London, London, United Kingdom; Instituto de Higiene e Medicina Tropical, Portugal

## Abstract

Family based association study (FBAS) has the advantages of controlling for population stratification and testing for linkage and association simultaneously. We propose a retrospective multilevel model (rMLM) approach to analyze sibship data by using genotypic information as the dependent variable. Simulated data sets were generated using the simulation of linkage and association (SIMLA) program. We compared rMLM to sib transmission/disequilibrium test (S-TDT), sibling disequilibrium test (SDT), conditional logistic regression (CLR) and generalized estimation equations (GEE) on the measures of power, type I error, estimation bias and standard error. The results indicated that rMLM was a valid test of association in the presence of linkage using sibship data. The advantages of rMLM became more evident when the data contained concordant sibships. Compared to GEE, rMLM had less underestimated odds ratio (OR). Our results support the application of rMLM to detect gene-disease associations using sibship data. However, the risk of increasing type I error rate should be cautioned when there is association without linkage between the disease locus and the genotyped marker.

## Introduction

The identification of single-nucleotide polymorphism (SNP) associated with complex diseases is an important goal of current genetic studies. Two important designs are commonly used: one recruits families (family-based association study, FBAS), and the other uses unrelated individuals (population-based association study) [1]. In terms of statistical power, the differences between the two methods are generally small when the disease is common or the minor allele frequency (MAF) is low [1], [2]. FBAS has the advantages of controlling for the effect of population stratification and can be used to test the hypothesis of both linkage and association. FBAS has also been used in genome-wide association studies (GWAS) and whole-genome sequencing researches [3], [4].

Several statistical methods have been proposed to analyze data from FBAS. Transmission disequilibrium test (TDT) is used to analyze case-parent trios data [5]. Sib transmission/disequilibrium test (S-TDT) can be used to analyze case-sibling data when parents' genetic information is unavailable in studying late-onset diseases [6]. Sibling disequilibrium test (SDT) can use the information from large sibships containing more than one affected individual [7]. However, neither of S-TDT and SDT is capable of adjusting for covariates, such as environmental exposures, gender, age, etc. Conditional logistic regression (CLR) is then a widely accepted method as it can include covariates [8].

S-TDT, SDT and CLR all require discordant sibships (DSSs) with at least one affected and one unaffected sibling. Thus the sibships with all siblings affected (namely, concordant sibships, CSSs) would be discarded, leading to a loss of information. Hancock et al. compared generalized estimation equations (GEE) to CLR using simulated family data [9]. Their findings showed that GEE can incorporate the information of CSSs, thus increasing the power to detect associations and gene-environmental interactions.

Multilevel model (MLM) could be a powerful tool for analyzing sibship data, as sibships collected from FBAS are featured by multilevel structure [10], [11]. Individuals, say, the first level units, are clustered within sibships (the second level units). It is also possible that sibships are nested in higher level units, such as communities or hospitals. The present study aims to examine some basic statistical properties of MLM for sibship data in comparison with existing methods such as S-TDT, SDT, CLR and GEE. The examination will focus on 10 scenarios based on simulated sibship datasets. Pros and cons of different methods will be discussed with recommendation of methodology.

## Methods

### Multilevel Logistic Model

Suppose the data consist of *n* ascertained sibships and each sibship has at least one affected individual. We use *i* = 1,2,..,*n* to denote the *i*th sibship, *j* = 1,2,…*J* to denote the *j*th individual in each sibship. When *J* = 2, the sibship becomes a sib pair. The corresponding two-level multilevel logistic model for the probability of being affected conditional on genetic information and other covariates is
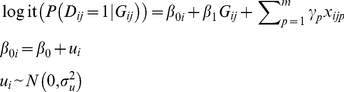
(1)Here, *D_ij_* = 1 denotes that the *j*th individual in the *i*th sibship is affected. *G_ij_* = 1 indicates that the corresponding individual carries the minor allele at the locus under study and *β*_1_ is the regression coefficient of *G_ij_* for the association between the disease and genetic marker. The intercept term is denoted by *β*_0_. The regression coefficients corresponding to environmental or demographic covariates are denoted by *γ*_1_,*γ*_2_,… and *γ_p_*. The *β*s and *γ*s are always referred to as the “fixed” parameters. The additional random effect, *u_i_*, denotes the residual in the second level, which is assumed to be a Gaussian distribution variable with a mean of zero. Its variance, 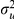, the “random” parameter, measures the variation among the sibships. A larger variance indicates a greater clustering effect or a stronger dependence within the sibships.

The exponential of *β*_1_ is the odds ratio (OR), an estimate of the genetic relative risk (GRR) when the disease prevalence is low. The Wald test can be used to test the hypothesis of *β*_1_ = 0 [10]. Parameters of multilevel logistic model can be estimated using an iteratively generalized least square method (IGLS) with Taylor series expansions and marginal/penalized quasi-likelihood (MQL/PQL) [12]. A simple improvement on IGLS gives restricted IGLS (RIGLS), which will produce unbiased variance estimate if sample size is limited. Goldstein and his colleagues developed the MLwiN software package for multilevel modeling [13]. MLM can also be fitted using other software packages, including SAS, Stata, S-Plus, HLM and R. However, MLwiN is recommended because of its high efficiency, friendly GUI and powerful macro languages [12].

It is also important to notice the difference between MLM and GEE [14], [15]. GEE estimates the marginal effect and produces robust variance estimates of the regression coefficients by taking the intra-cluster correlation into consideration. It specifies a working correlation matrix for the data and treats the intra-cluster correlation as nuisance. MLM estimates cluster-specific effects by including an random term to vary with clusters. With the ability to including random intercept or coefficients, MLM can model data with complex hierarchical structures. The interpretation of OR estimates derived from GEE and MLM are also different. In GEE, OR can be interpreted as the odds of affected from a population with the risk genotype compared to that from a population without the risk genotype. As MLM adjusts for the heterogeneity among subjects, it produces OR estimates which represents the change on odds of affected due to the genotypes for a single person (say, the odds of affected from a person with the risk genotype compared to that from the SAME person if he does not carry the risk genotype.)

### Retrospective Multilevel Logistic Model

However, in family based studies, it is common practice for the samples to be collected using a proband-ascertained method, by which the families are sampled through an affected member (proband) [16]. All sibships in the sample would have at least one affected individual. This may result in a high degree of within-family correlation and a low degree of among-family variation. Since the variation among sibships would be almost impossible to extract from the total variation, the MLM would degenerate to an ordinary unconditional logistic regression model, as shown by the results of our simulations. In order to resolve this problem, a retrospective multilevel logistic model (rMLM) is proposed.

RMLM uses genotypic information as the dependent variable and disease status as the independent variable. A retrospective two-level logistic model for the probability of carrying the minor allele is
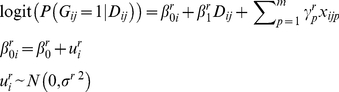
(2)In equation (2), the “*r*” in the superscripts indicates that the corresponding coefficient is from a retrospective model. It is not difficult to show that 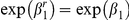 [17].

### Retrospective Generalized Estimation Equation

The idea of “retrospective” modeling can be easily applied to GEE [18], which leads to retrospective GEE (rGEE). For simplicity, we use GEEi, GEEe, rGEEi and rGEEe to denote GEE with an independent, GEE with an exchangeable, retrospective GEE with an independent and retrospective GEE with an exchangeable working correlation matrix, respectively.

### Data Simulation

Simulated data sets were generated using the simulation of linkage and association (SIMLA) software (V3.3) [19]. For each data set in our simulation, we considered 4 standard hypotheses in genetic association studies through the different relationships among 4 markers (M1 to M4) and a disease locus (D) on two simulated chromosomes. M1 was both linked and associated with D, corresponding to the alternative hypothesis. The three null hypotheses were linkage but no association (as simulated by M2), association but no linkage (M3) and no linkage and no association (M4). D, M1, M2 and M3 were all on the first chromosome. The distances from D to M1, M2 and M3 were 0.01 cM (centimorgan), 0.01 cM and 5 Morgan, respectively. M4 was on the different chromosome other than D. Perfect linkage disequilibrium (LD) was simulated between M1 and D with *r* ^2^ = 1, while moderate LD (*r* ^2^ = 0.26) was simulated between M3 and D. The disease allele frequency (DAF) of D was fixed at 0.20. We specified the minor allele frequencies (MAFs) of M1 and M3 to be 0.20 and 0.32, respectively. Both MAFs of M2 and M4 were specified to be 0.5. For simplicity, we considered a dominant genetic model for all the scenarios. The prevalence of the disease was fixed at 5%.

Ten scenarios were considered in our simulations. For each setting in each scenario, we simulated 1,000 replicates. In order to generate the sibships which the data sets were comprised of, we simulated three types of pedigrees, denoted by A, B and C, respectively, by using three ascertainment criteria embedded in SIMLA: proband, affected cousin pairs and at least one affected sib pair. The size of sibship was set at 2 in most scenarios, except for scenario 10, in which the sibships from 100 pedigrees (10%) had 3 members. Specified numbers of pedigrees A, B and C were then pooled, as shown by Table 1. Data from the grandparental and parental generations were removed to reflect infeasible ascertainment of older generations. For sibships in the latest generation, only those with at least one affected individual were retained. In scenarios 1–8, “hypothesized” proportion of discordant sib pairs (DSPs) was defined as the proportion of pedigree A, which approximately determined the proportion of DSPs in the simulated data set. The hypothesized proportion of concordant sib pairs (CSPs) was defined as one minus the hypothesized proportion of DSPs. We named them “hypothesized” proportion because the proband ascertainment did not necessarily ensure the simulated data sets consisting only of DSPs. Thus even in scenarios 1 and 5 with hypothesized proportions of DSPs being 100%, the actual proportions of CSPs were both around 5%. In scenarios 4 and 8 with hypothesized 70% of DSPs, the actual proportions of CSPs were around 32%.

**Table 1:** Parameter settings of the 10 scenarios.

	Pedigree Type#				
Scenario	A§	B	C	Genetic Relative Risk	Environmental Relative Risk
1	1000	0	0	1.5;2	-
2	900	0	100	1.5;2	-
3	800	0	200	1.5;2	-
4	700	0	300	1.5;2	-
5	1000	0	0	1.5	1.5
6	900	0	100	1.5	1.5
7	800	0	200	1.5	1.5
8	700	0	300	1.5	1.5
9	900	100	0	1.5	1.5
10*	900A2+100A3	0	0	1.5	1.5

# A, B and C denote pedigrees ascertained by proband, affected cousin pair and at least one affected sib pair, respectively.

§ In scenarios 1–8, the proportion of pedigree A (“hypothesized” proportion of DSPs) approximately determined the proportion of discordant sib pairs in the simulated data set.

*In scenario 10, pedigrees were all ascertained by proband. However, 900 of the 1000 pedigrees had the sibships' size of 2, while the other 100 pedigrees had the size of 3.

We only considered the single gene disease model in our simulation. The first 8 scenarios were designed to simulate the situations when there were specified hypothesized proportions of CSPs in the data sets. In scenarios 1–4, data sets were simulated with a GRR of the disease locus of either 1.5 or 2. In scenarios 5–8, the GRR was fixed at 1.5, and a continuous environmental factor was also simulated with a relative risk (RR) of 1.5 to examine the performance of each method when there was heterogeneity among families due to some environmental exposure in familial aggregation. We assumed the exposure was a random variable sampled from a Gaussian distribution, with a within-pedigree correlation of 0.5. We considered two special situations in the last two scenarios. Scenario 9 simulated the data sets with dependent sibships by including pedigrees of type B. In scenario 10, 100 pedigrees were designed to have sibships with 3 individuals to reflect the situation when there were various sizes of sibships in the data. Just like scenarios 5–8, an environmental factor correlated within pedigrees was also simulated in each of the last two scenarios.

### Statistical Analysis

MLwiN (version 2.13, Bristol, UK) was used to perform MLM and rMLM analysis. Two-level logistic regression models were used to fit the simulated data, with individuals as the first and sibships as the second level units. Although an environmental factor was simulated in scenario 5–10, it was not included in the model to reflect an unknown/un-measurable factor within family. Parameters were estimated using RIGLS with a 2^nd^-order linearization and PQL approach. A sample code for rMLM modeling can be found in Text S1.

The SAS system (Version 9.1.3, Cary, NC) was used for the other analyses. The ***Family*** procedure was used to implement S-TDT and SDT, the ***PHReg*** procedure was used to implement CLR with a robust variance estimate to evaluate the significance [8]. The ***GENMOD*** procedure was used to implement GEE and rGEE.

Basic statistical properties of each method were examined in terms of power, type I error, estimation bias and standard error. For every 1,000 replicates, type I error rate was calculated as the proportion of rejecting the null hypothesis in the model with unlinked and/or un-associated marker (M2, M3 or M4). Power was calculated as the proportion of rejecting the null hypothesis in the model with linked and associated marker (M1). We computed the average odds ratio (OR) using 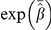, in which 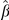 is the regression coefficient of the marker in MLM, CLR, GEEi or GEEe, and of the disease status in rMLM, rGEEi or rGEEe. Estimation bias was examined by comparing the average OR to the corresponding true GRR defined in our simulations. We also computed the empirical standard error over the 1,000 replicates to measure the variation of the estimates. Limits of empirical 95% CI were estimated as the 2.5th and 97.5th percentiles of the OR estimates from the 1,000 replicates.

## Results

### Influence of concordant sibships

**Figure 1:**
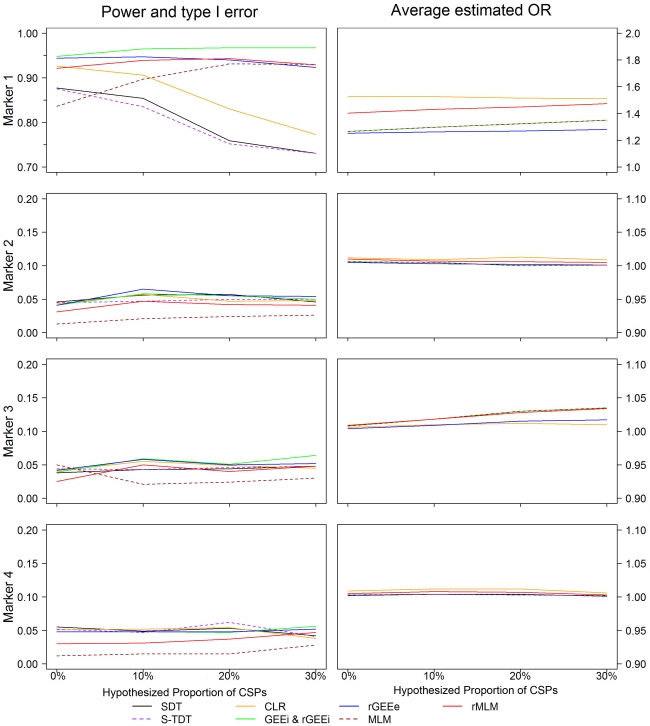
Measures of power (M1), type I error (M2) and parameter estimate of scenarios 1 to 4. The left panel of the figure shows the measures of power and type I error rate of rMLM, MLM, S-TDT, SDT, CLR, GEEi, rGEEi and rGEEe when there were different hypothesized proportions of CSPs in the data sets in scenarios 1 to 4 with GRR = 1.5. The right panel shows the parameter estimates of rMLM, MLM, CLR, GEEi, rGEEi and rGEEe. In each plot, the *x*-axis denotes the hypothesized proportions of CSPs (one minus the hypothesized proportions of DSPs). As GEEi and rGEEi had the same results throughout our simulation, they are represented by the same color. GEEe is not shown due to its unstable results.

Figure 1 shows the results of power, type I error rate and parameter estimate when GRR = 1.5 for scenarios 1–4, in which the hypothesized proportion of CSPs are 0%,10%, 20% and 30%, respectively. Full descriptions of the results are listed in Table S1 and Table S2.

For the hypothesis of both linkage and association (M1), S-TDT, SDT and CLR were all valid methods if almost all sib pairs were discordant. Power from the three methods was around 80% or 100% when GRR = 1.5 or 2.0. However, their powers were reduced by the increasing proportion of CSPs. When GRR = 1.5 and the hypothesized proportion of CSPs was 30% (namely, the hypothesized proportion of DSPs was 70%), none of S-TDT, SDT and CLR had power greater than 80%. In contrast, rMLM, GEEi, rGEEi and rGEEe had almost the same level of power, which was greater than 90%, almost independent of the proportion of CSPs. The OR estimates of M1 by rMLM, GEEi, rGEEi and rGEEe were lower than the true GRR and those by CLR, while the degree of the underestimate became less with the increasing proportion of CSPs. By comparing the average OR estimates of M1 in Figure 1 and Table S2 to the true GRRs, we found that rMLM was the least biased among the three retrospective methods. OR estimates from CLR were more variable than those from the other methods, as shown by the empirical estimates of the standard error of OR in Table S2.

Type I error rates were compared with the results of M2, M3 and M4. For M2 and M4, all methods protected the type I error rate at the significant level of 0.05. However, for M3, the type I error rates of both GEEi and rGEEi showed trends of slightly inflating with the increasing proportion of CSPs. The degree of inflation became prominent when GRR = 2.0, as shown by Table S1. Although rMLM and rGEEe both slightly overestimated OR of M3, their type I error rates were lower than GEEi and rGEEi, even when GRR = 2.0 and the hypothesized proportion of CSPs was 30%.

Conclusions derived from scenarios 5–8 (Table S3 and Table S4) are very similar to those from scenarios 1–4. The powers of rMLM were all greater than 90%, with less underestimated parameters than those of GEE and rGEE. Additionally, rMLM was less likely to have inflated type I error on M3 than rGEE would. Again, CSPs in the simulated data sets reduced the power and increased the variation of the OR estimates of CLR.

Results from GEEi and rGEEi were numerically identical throughout our simulation. They are represented by the same color in Figure 1 and summarized in the same column in Tables 2 and S1, S2,S3,S4, and S5. Their results were the same even when a discrete or continuous environmental factor was included as a fixed effect in the model (not shown in this article). GEEe had the most unstable results with reduced power on M1 and inflated type I error on M3 under most scenarios. Although MLM had the same OR estimates as GEEi, its power was much lower. The estimates of 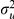 of MLM in all scenarios were 0 s, indicating that the multilevel logistic models in the simulations degenerated to standard unconditional logistic regression models.

**Table 2:** Measures of power (M1), type I error (M2–M4) and parameter estimate (average OR, empirical standard error and 95%CI) of scenario 9 in which the simulated datasets contain affected cousin pairs.

	Marker	S-TDT	SDT	CLR	MLM	GEEe	GEEi & rGEEi	rGEEe	rMLM
Power and type I error rate	M1	0.839	0.850	0.900	0.925	0.781	0.973	0.957	0.946
	M2	0.044	0.046	0.053	0.019	0.053	0.053	0.055	0.036
	M3	0.051	0.049	0.058	0.024	0.069	0.056	0.060	0.057
	M4	0.059	0.057	0.043	0.012	0.048	0.048	0.045	0.036
Parameter estimation	M1	-	-	1.51±0.19	1.30±0.09	1.14±0.05	1.30±0.09	1.26±0.08	1.45±0.14
		-	-	(1.18,1.93)	(1.14,1.48)	(1.03,1.24)	(1.14,1.48)	(1.12,1.42)	(1.20,1.75)
	M2	-	-	1.01±0.14	1.00±0.08	1.00±0.05	1.00±0.08	1.00±0.08	1.00±0.11
		-	-	(0.76,1.29)	(0.86,1.17)	(0.90,1.11)	(0.86,1.17)	(0.86,1.16)	(0.81,1.23)
	M3	-	-	1.01±0.12	1.02±0.07	1.03±0.05	1.02±0.07	1.01±0.07	1.02±0.10
		-	-	(0.79,1.28)	(0.89,1.17)	(0.94,1.12)	(0.89,1.17)	(0.88,1.15)	(0.83,1.24)
	M4	-	-	1.01±0.13	1.00±0.07	1.00±0.05	1.00±0.07	1.00±0.07	1.00±0.10
		-	-	(0.78,1.27)	(0.88,1.14)	(0.92,1.10)	(0.88,1.14)	(0.88,1.14)	(0.82,1.22)

### Dependent sibships and sibships with various sizes

The results from scenarios 9–10 are presented in Table 2 and Table S5, respectively. RMLM continued to gain more power than CLR, SDT and S-TDT. When compared to GEEi, rGEEi and rGEEe, although rMLM had slightly lower power, it had less underestimated OR.

## Discussion

To test for the association using sibship data, traditional methods require data consisting of DSPs or DSSs [6], [7], [20]. Both GEE and MLM are designed for data with clustering effects, and can handle data with both DSSs and CSSs in theory. Previous study has already showed the advantage of GEE over CLR [9]. The present study advances the methodology field further by showing that MLM, comparable to GEE in many aspects, is a valid approach with good statistical properties in analyzing sibship data. We propose to analyze the sibship data by a retrospective multilevel model (rMLM) which estimates the genotypic outcome conditional on the disease status. Simulations showed that rMLM and GEE gained more power than SDT, S-TDT and CLR when the data set contained CSSs. The increased power of rMLM is likely due to the enlarged effective sample size. Both of rMLM and GEE can “borrow” information across sibships, thus have the advantage of utilizing information from CSSs by comparing cases to their sibling controls and to controls across the population. In contrast, traditional methods compare cases only to their sibling controls and sibships with all members affected will be ignored. Our simulations also demonstrated that rMLM had increased power than SDT, S-TDT and CLR when the data set contained dependent sibships or sibships with various sizes. Compared to GEE and rGEE, rMLM is preferable. Although its power was slightly lower, rMLM had parameter estimates much closer to the true GRR defined in our simulations.

GEE, rGEE and rMLM all produced negatively biased OR estimates of genetic effect in our simulation, and the degree of bias was greater when the genetic effect was large. There are several issues needed to be clarified on this bias. Firstly, none of the OR estimates resulting from these methods, including CLR, GEE, rGEE, MLM and rMLM, may be reliably interpreted as accurate estimates of GRR in the population. OR is an approximation of RR only when the disease is rare. Secondly, the ascertainment procedure may be inadequately modeled. The sample does not represent the general population but the ascertained one [21]. An ascertained population is a subset of the general population and is comprised of all the families in the general population with at least one affected individual. Retrospective modeling takes the ascertainment procedure into consideration by using disease status as the predictor, thus providing estimates closer to the true parameters than those using disease status as the outcome. Thirdly, rMLM gave less biased estimates than rGEEi and rGEEe. The reason is that GEE is a population average (PA) method, while MLM is a cluster-specified method [14], [22]. In most situations, the former is closer to the null hypothesis, and it would be more apparent when the within-family correlation is high [23].

Our simulations indicated that rMLM, GEE and rGEE all gained more power than CLR with the increasing proportion of CSPs. It may be possible that there are more than 5% of CSPs in the ascertained sample. As an example, the prevalence of total diabetes and prediabetes in China is about 9.7% [24]. Thus theoretically, the possibility that two individuals from the same sib pair are both affected is 0.94%. Due to the fact that a sib pair would be sampled only if it has at least one affected member, the proportion of CSPs in the sample would be about 5.10%, a figure very close to scenario 1 and 5 in our simulations. And the proportion of CSPs will be greater in some behavior genetic researches. Even in the most “extreme” situation in which all the CSPs were removed in scenario 1, the power of rMLM was only slightly lower than CLR (87% *vs* 90%, not shown in this article). Given that rMLM provides less biased estimates than GEE and rGEE and is more flexible for modeling complex hierarchical structures, we believe that rMLM has practical value.

However, when the markers and the disease locus were under association without linkage (M3), both rMLM and rGEE seemed to have inflated type I error rates, especially when GRR = 2.0. M3 is an extreme example of population stratification. To achieve this level, allele frequency and disease prevalence between sub-populations should be “drastically” different [9], [25]. Although the type I error rate of rMLM was only slighted inflated when genetic effect is modest, we should still be cautious to use rMLM in these situations. If there is prior information that does suggest the existence of population stratification, involving the stratification factor as covariates should be considered. If the chip used in the study has a large number of markers or contains ancestry information markers (AIMs), some powerful methods have been proposed to detect and adjust for population stratification, such as genomic control [26] and EIGENSTRAT [27]. Hinrichs et al. showed that genetically related individuals may induce bias to the decomposition of principal components in EIGENSTRAT analysis under certain circumstances (e.g., small sample), and they suggested to use a weighted principal component analysis (PCA) under these conditions [28]. Another possible solution is to use a subset of unrelated individuals sampled from the overall sample to extract the information on population stratification.

This study has several limitations. Firstly, we only used the dominant genetic model in our simulations. Within MLM framework using MLwiN, we can in theory fit a retrospective multilevel ordinal logistic model for additive genetic model, or a retrospective multilevel multinomial logistic model for co-dominant genetic model. However, some methodological issues need clarification. The coefficients of retrospective ordinal logistic model may not be easy to explain. Equations to convert them to gene's ORs may not be as straightforward as that in retrospective logistic model for dichotomous outcome. Meanwhile, multilevel ordinal logistic model may fail to converge if small proportion presents in some category of the outcome. A possible solution is using the allele instead of the genotype as the outcome in the first level and treating individuals as the second level units. It could “produce” more data for the outcome and may increase the chance of convergence. This model can also account for the correlation between alleles. Secondly, retrospective models may have difficulties in handling the simultaneous effects of several markers. One possible solution for this is to use cumulative polygenetic effects as the outcome. Under the multilevel framework, we also think it is possible to simultaneously treat the SNPs as outcomes by using a multiple responses multilevel model [29].

With recent advancements in genotyping technologies, it is possible to genotype thousands, even millions of SNPs simultaneously. We also notice the potential value of rMLM in analyzing disease-SNP set association, which is an important issue in gene-based and pathway-based analysis [30], [31], even when data sets are from population-based case control studies. In the retrospective framework, SNP information from the same individual is on the left side of the equation, thus making it possible to use the covariance to account for the LD among SNPs. However, further investigation is needed to clarify the application of MLM in these fields.

## Supporting Information

Text S1 Sample Code for fitting a retrospective multilevel model using MLwiN Macro.
(DOC)

Table S1 Measures of power (M1) and type I error (M2–M4) of scenarios 1–4.
(DOC)

Table S2 Measures of parameter estimates (average OR, empirical standard error and 95%CI) of scenarios 1–4.
(DOC)

Table S3 Measures of power (M1) and type I error (M2–M4) of scenarios 5–8.
(DOC)

Table S4 Measures of parameter estimates (average OR, empirical standard error and 95%CI) of scenarios 5–8.
(DOC)

Table S5 Measures of power (M1), type I error (M2–M4) and parameter estimate (average OR, empirical standard error and 95%CI) of scenario 10.
(DOC)
