# Supplementary material for: Genetic Association Analysis Using Sibship Data: A Multilevel Model Approach
Source: PLoS One. 2012 Feb 1;7(2):e31134. doi: 10.1371/journal.pone.0031134 (PMC3270036; doi:10.1371/journal.pone.0031134)
Supplement: Text S1 — Sample Code for fitting a retrospective multilevel model using MLwiN Macro. (DOC) [file pone.0031134.s001.doc]

**Supporting Information**

**Text S1. Sample Code for fitting a retrospective multilevel model using MLwiN Macro.**

| DINP c1-c13 | #read data from disk |
| --- | --- |
| H:\ \Data\ML\1.txt |  |
| Name C1 'Famid' C2 'id' C6 'dis' | #name the columns |
| Name C7 'marker1' |  |
| Name C8 'marker2' |  |
| Name C9 'marker3' |  |
| Name C10 'marker4' |  |
| Name C11 'famsib' |  |
| Name C12 'Cons' |  |
| Name C13 'Denom' |  |
| Resp 'marker2' | #set "M2" to be the outcome |
| rdist 1 0 | #specify outcome's distribution ("binomial") |
| lfun 0 | #specify link function ("logit") |
| linea 1 2 | #specify 2nd PQL to be the approximation procedure |
| doffs 1 'denom' |  |
| iden 2 'famid' | #specify level 2 unit |
| iden 1 'id' | #specify level 1 unit |
| addt 'cons' | #add intercept in the model |
| setv 2 'cons' | #specify random effect |
| addt 'Dis' | #add disease status as the covariate in the retrospective model |
| meth 0 | #use RIGLS for model estimation |
| batch 1 | #batch mode on |
| maxi 100 | #set maximum iteration time to be 100 |
| star | #start iteration |
| next | #more iterations to ensure convergence |
