# Supplementary material for: Genetic Association Analysis Using Sibship Data: A Multilevel Model Approach
Source: PLoS One. 2012 Feb 1;7(2):e31134. doi: 10.1371/journal.pone.0031134 (PMC3270036; doi:10.1371/journal.pone.0031134)
Supplement: Table S1 — Measures of power (M1) and type I error (M2–M4) of scenarios 1–4. (DOC) [file pone.0031134.s002.doc]

**Supporting Information**

**Table S1. Measures of power (M1) and type I error (M2-M4) of scenarios 1-4.**

| Scenario | GRR | Marker | S-TDT | SDT | CLR | GEEe | GEEi  &rGEEi | rGEEe | MLM | rMLM |
| --- | --- | --- | --- | --- | --- | --- | --- | --- | --- | --- |
| 1 | 1.5 | M1 | 0.875 | 0.877 | 0.926 | 0.356 | 0.948 | 0.944 | 0.836 | 0.921 |
| Hypothesized proportion of DSPs=1.0 |  | M2 | 0.045 | 0.046 | 0.043 | 0.055 | 0.041 | 0.041 | 0.013 | 0.031 |
|  |  | M3 | 0.044 | 0.038 | 0.041 | 0.048 | 0.039 | 0.042 | 0.050 | 0.025 |
|  |  | M4 | 0.052 | 0.055 | 0.051 | 0.056 | 0.048 | 0.048 | 0.012 | 0.030 |
|  | 2.0 | M1 | 0.999 | 0.999 | 1.000 | 0.826 | 1.000 | 1.000 | 1.000 | 1.000 |
|  |  | M2 | 0.056 | 0.061 | 0.058 | 0.054 | 0.054 | 0.059 | 0.015 | 0.043 |
|  |  | M3 | 0.057 | 0.050 | 0.053 | 0.074 | 0.046 | 0.052 | 0.011 | 0.034 |
|  |  | M4 | 0.042 | 0.042 | 0.046 | 0.045 | 0.045 | 0.041 | 0.009 | 0.028 |
| 2 | 1.5 | M1 | 0.835 | 0.854 | 0.906 | 0.707 | 0.965 | 0.947 | 0.897 | 0.939 |
| Hypothesized proportion of DSPs=0.9 |  | M2 | 0.047 | 0.056 | 0.057 | 0.056 | 0.058 | 0.065 | 0.021 | 0.047 |
|  |  | M3 | 0.043 | 0.043 | 0.055 | 0.072 | 0.059 | 0.058 | 0.021 | 0.050 |
|  |  | M4 | 0.047 | 0.049 | 0.052 | 0.044 | 0.048 | 0.048 | 0.015 | 0.031 |
|  | 2.0 | M1 | 1.000 | 1.000 | 1.000 | 0.995 | 1.000 | 1.000 | 1.000 | 1.000 |
|  |  | M2 | 0.040 | 0.044 | 0.046 | 0.057 | 0.054 | 0.050 | 0.022 | 0.034 |
|  |  | M3 | 0.061 | 0.059 | 0.052 | 0.121 | 0.069 | 0.063 | 0.027 | 0.051 |
|  |  | M4 | 0.036 | 0.041 | 0.031 | 0.051 | 0.046 | 0.037 | 0.015 | 0.031 |
| 3 | 1.5 | M1 | 0.752 | 0.759 | 0.830 | 0.861 | 0.967 | 0.940 | 0.931 | 0.943 |
| Hypothesized proportion of DSPs=0.8 |  | M2 | 0.050 | 0.057 | 0.047 | 0.051 | 0.055 | 0.055 | 0.024 | 0.042 |
|  |  | M3 | 0.046 | 0.044 | 0.049 | 0.076 | 0.051 | 0.050 | 0.024 | 0.040 |
|  |  | M4 | 0.062 | 0.053 | 0.054 | 0.041 | 0.047 | 0.048 | 0.015 | 0.037 |
|  | 2.0 | M1 | 0.997 | 0.997 | 1.000 | 1.000 | 1.000 | 1.000 | 1.000 | 1.000 |
|  |  | M2 | 0.061 | 0.050 | 0.050 | 0.033 | 0.053 | 0.060 | 0.021 | 0.045 |
|  |  | M3 | 0.050 | 0.053 | 0.049 | 0.137 | 0.086 | 0.059 | 0.041 | 0.058 |
|  |  | M4 | 0.052 | 0.048 | 0.048 | 0.043 | 0.043 | 0.050 | 0.017 | 0.039 |
| 4 | 1.5 | M1 | 0.731 | 0.731 | 0.773 | 0.925 | 0.967 | 0.923 | 0.930 | 0.929 |
| Hypothesized proportion of DSPs=0.7 |  | M2 | 0.050 | 0.046 | 0.048 | 0.052 | 0.050 | 0.054 | 0.026 | 0.041 |
|  |  | M3 | 0.048 | 0.048 | 0.045 | 0.076 | 0.064 | 0.052 | 0.030 | 0.048 |
|  |  | M4 | 0.041 | 0.042 | 0.038 | 0.060 | 0.056 | 0.052 | 0.028 | 0.047 |
|  | 2.0 | M1 | 0.989 | 0.994 | 0.998 | 1.000 | 1.000 | 1.000 | 1.000 | 1.000 |
|  |  | M2 | 0.053 | 0.058 | 0.046 | 0.049 | 0.059 | 0.051 | 0.027 | 0.037 |
|  |  | M3 | 0.047 | 0.047 | 0.042 | 0.141 | 0.093 | 0.059 | 0.059 | 0.058 |
|  |  | M4 | 0.055 | 0.046 | 0.051 | 0.052 | 0.048 | 0.053 | 0.024 | 0.040 |
