# Supplementary material for: Genetic Association Analysis Using Sibship Data: A Multilevel Model Approach
Source: PLoS One. 2012 Feb 1;7(2):e31134. doi: 10.1371/journal.pone.0031134 (PMC3270036; doi:10.1371/journal.pone.0031134)
Supplement: Table S2 — Measures of parameter estimates (average OR, empirical standard error and 95%CI) of scenarios 1–4. (DOC) [file pone.0031134.s003.doc]

**Supporting Information**

**Table S2. Measures of parameter estimates (average OR, empirical standard error and 95%CI) of scenarios 1-4.**

| Scenario | GRR | Marker | CLR | GEEe | GEEi  &rGEEi | rGEEe | MLM | rMLM |
| --- | --- | --- | --- | --- | --- | --- | --- | --- |
| 1 | 1.5 | M1 | 1.53±0.19 | 1.05±0.03 | 1.27±0.08 | 1.25±0.08 | 1.27±0.08 | 1.40±0.13 |
| Hypothesized proportion of DSPs=1.0 |  |  | (1.20,1.94) | (0.99,1.12) | (1.11,1.44) | (1.11,1.42) | (1.11,1.44) | (1.17,1.69) |
|  |  | M2 | 1.01±0.13 | 1.00±0.035 | 1.01±0.08 | 1.01±0.08 | 1.01±0.08 | 1.01±0.12 |
|  |  |  | (0.79,1.31) | (0.93,1.07) | (0.87,1.17) | (0.87,1.17) | (0.87,1.17) | (0.82,1.26) |
|  |  | M3 | 1.01±0.12 | 1.01±0.03 | 1.01±0.06 | 1.00±0.06 | 1.01±0.06 | 1.01±0.09 |
|  |  |  | (0.81,1.26) | (0.96,1.07) | (0.89,1.14) | (0.89,1.14) | (0.89,1.14) | (0.85,1.21) |
|  |  | M4 | 1.01±0.13 | 1.00±0.03 | 1.00±0.07 | 1.00±0.07 | 1.00±0.07 | 1.01±0.10 |
|  |  |  | (0.78,1.28) | (0.94,1.07) | (0.88,1.14) | (0.88,1.13) | (0.88,1.14) | (0.82,1.21) |
|  | 2 | M1 | 2.02±0.26 | 1.09±0.04 | 1.48±0.10 | 1.45±0.09 | 1.48±0.10 | 1.75±0.17 |
|  |  |  | (1.55,2.60) | (1.02,1.17) | (1.29,1.67) | (1.28,1.65) | (1.29,1.69) | (1.45,2.13) |
|  |  | M2 | 1.01±0.14 | 1.00±0.04 | 1.00±0.08 | 1.00±0.08 | 1.00±0.08 | 1.01±0.12 |
|  |  |  | (0.76,1.32) | (0.93,1.07) | (0.86,1.17) | (0.86,1.17) | (0.86,1.17) | (0.79,1.27) |
|  |  | M3 | 1.00±0.12 | 1.02±0.03 | 1.01±0.07 | 1.00±0.07 | 1.01±0.07 | 1.01±0.09 |
|  |  |  | (0.78,1.25) | (0.96,1.07) | (0.88,1.14) | (0.88,1.13) | (0.88,1.14) | (0.83,1.20) |
|  |  | M4 | 1.02±0.13 | 1.00±0.03 | 1.01±0.07 | 1.01±0.07 | 1.01±0.07 | 1.01±0.10 |
|  |  |  | (0.79,1.30) | (0.94,1.07) | (0.89,1.14) | (0.88,1.14) | (0.89,1.14) | (0.83,1.22) |
| 2 | 1.5 | M1 | 1.53±0.19 | 1.13±0.06 | 1.30±0.09 | 1.26±0.08 | 1.30±0.09 | 1.43±0.13 |
| Hypothesized proportion of DSPs=0.9 |  |  | (1.18,1.94) | (1.03,1.25) | (1.14,1.47) | (1.11,1.42) | (1.14,1.47) | (1.18,1.71) |
|  |  | M2 | 1.01±0.15 | 1.00±0.06 | 1.01±0.09 | 1.00±0.08 | 1.01±0.09 | 1.01±0.13 |
|  |  |  | (0.75,1.34) | (0.89,1.12) | (0.85,1.19) | (0.85,1.18) | (0.85,1.19) | (0.78,1.28) |
|  |  | M3 | 1.01±0.13 | 1.02±0.05 | 1.02±0.07 | 1.01±0.07 | 1.02±0.07 | 1.02±0.10 |
|  |  |  | (0.78,1.28) | (0.93,1.12) | (0.88,1.17) | (0.88,1.15) | (0.88,1.17) | (0.83,1.23) |
|  |  | M4 | 1.01±0.13 | 1.00±0.05 | 1.00±0.07 | 1.00±0.07 | 1.00±0.07 | 1.01±0.10 |
|  |  |  | (0.78,1.30) | (0.91,1.11) | (0.87,1.15) | (0.88,1.14) | (0.87,1.15) | (0.82,1.22) |
|  | 2 | M1 | 2.02±0.28 | 1.24±0.06 | 1.55±0.11 | 1.48±0.10 | 1.55±0.11 | 1.81±0.18 |
|  |  |  | (1.54,2.62) | (1.13,1.36) | (1.35,1.78) | (1.30,1.68) | (1.35,1.78) | (1.50,2.18) |
|  |  | M2 | 1.00±0.14 | 1.00±0.06 | 1.00±0.08 | 1.00±0.08 | 1.00±0.08 | 1.00±0.12 |
|  |  |  | (0.76,1.31) | (0.89,1.12) | (0.84,1.17) | (0.85,1.16) | (0.84,1.17) | (0.78,1.25) |
|  |  | M3 | 1.00±0.13 | 1.04±0.05 | 1.03±0.07 | 1.01±0.07 | 1.03±0.07 | 1.03±0.10 |
|  |  |  | (0.77,1.28) | (0.94,1.14) | (0.89,1.18) | (0.88,1.16) | (0.89,1.18) | (0.84,1.24) |
|  |  | M4 | 1.01±0.13 | 1.00±0.05 | 1.00±0.07 | 1.00±0.07 | 1.00±0.07 | 1.00±0.10 |
|  |  |  | (0.78,1.27) | (0.90,1.10) | (0.87,1.15) | (0.88,1.13) | (0.87,1.15) | (0.82,1.20) |
| 3 | 1.5 | M1 | 1.51±0.21 | 1.20±0.07 | 1.32±0.10 | 1.27±0.09 | 1.32±0.10 | 1.45±0.15 |
| Hypothesized proportion of DSPs=0.8 |  |  | (1.14,1.98) | (1.07,1.35) | (1.14,1.52) | (1.11,1.45) | (1.14,1.52) | (1.18,1.77) |
|  |  | M2 | 1.01±0.15 | 1.00±0.07 | 1.00±0.09 | 1.00±0.08 | 1.00±0.09 | 1.01±0.12 |
|  |  |  | (0.74,1.34) | (0.87,1.14) | (0.84,1.19) | (0.85,1.18) | (0.84,1.19) | (0.79,1.29) |
|  |  | M3 | 1.01±0.13 | 1.03±0.06 | 1.03±0.08 | 1.02±0.07 | 1.03±0.08 | 1.03±0.10 |
|  |  |  | (0.77,1.29) | (0.92,1.16) | (0.89,1.18) | (0.88,1.16) | (0.89,1.18) | (0.84,1.24) |
|  |  | M4 | 1.01±0.14 | 1.00±0.06 | 1.00±0.07 | 1.00±0.07 | 1.00±0.07 | 1.01±0.11 |
|  |  |  | (0.77,1.33) | (0.89,1.13) | (0.86,1.16) | (0.87,1.15) | (0.86,1.16) | (0.81,1.23) |
|  | 2 | M1 | 2.04±0.30 | 1.37±0.08 | 1.62±0.12 | 1.51±0.11 | 1.62±0.12 | 1.89±0.20 |
|  |  |  | (1.53,2.77) | (1.22,1.53) | (1.40,1.89) | (1.31,1.73) | (1.40,1.89) | (1.54,2.33) |
|  |  | M2 | 1.01±0.15 | 1.01±0.07 | 1.01±0.09 | 1.01±0.08 | 1.01±0.09 | 1.01±0.12 |
|  |  |  | (0.74,1.33) | (0.88,1.14) | (0.84,1.19) | (0.84,1.18) | (0.84,1.19) | (0.78,1.27) |
|  |  | M3 | 1.00±0.14 | 1.06±0.06 | 1.04±0.08 | 1.02±0.07 | 1.04±0.08 | 1.04±0.11 |
|  |  |  | (0.77,1.29) | (0.94,1.18) | (0.89,1.21) | (0.88,1.17) | (0.89,1.21) | (0.84,1.27) |
|  |  | M4 | 1.01±0.14 | 1.000±0.06 | 1.00±0.07 | 1.00±0.07 | 1.00±0.07 | 1.00±0.10 |
|  |  |  | (0.75,1.30) | (0.89,1.12) | (0.86,1.15) | (0.87,1.14) | (0.86,1.15) | (0.81,1.22) |
| 4 | 1.5 | M1 | 1.51±0.22 | 1.26±0.09 | 1.35±0.11 | 1.28±0.09 | 1.35±0.106 | 1.47±0.16 |
| Hypothesized proportion of DSPs=0.7 |  |  | (1.12,1.99) | (1.11,1.45) | (1.16,1.57) | (1.11,1.47) | (1.16,1.57) | (1.18,1.79) |
|  |  | M2 | 1.01±0.16 | 1.00±0.08 | 1.00±0.09 | 1.00±0.09 | 1.00±0.09 | 1.01±0.13 |
|  |  |  | (0.73,1.36) | (0.85,1.17) | (0.83,1.19) | (0.84,1.18) | (0.83,1.19) | (0.78,1.29) |
|  |  | M3 | 1.01±0.14 | 1.04±0.07 | 1.04±0.08 | 1.02±0.07 | 1.04±0.08 | 1.03±0.11 |
|  |  |  | (0.76,1.31) | (0.90,1.18) | (0.88,1.20) | (0.88,1.18) | (0.88,1.20) | (0.84,1.27) |
|  |  | M4 | 1.01±0.14 | 1.00±0.07 | 1.01±0.08 | 1.00±0.07 | 1.00±0.08 | 1.00±0.11 |
|  |  |  | (0.74,1.31) | (0.86,1.16) | (0.84,1.18) | (0.86,1.16) | (0.84,1.18) | (0.79,1.25) |
|  | 2 | M1 | 2.03±0.31 | 1.48±0.10 | 1.67±0.13 | 1.53±0.11 | 1.67±0.13 | 1.94±0.21 |
|  |  |  | (1.49,2.71) | (1.29,1.69) | (1.43,1.95) | (1.32,1.76) | (1.43,1.95) | (1.56,2.39) |
|  |  | M2 | 1.02±0.16 | 1.00±0.08 | 1.00±0.09 | 1.01±0.09 | 1.00±0.09 | 1.01±0.13 |
|  |  |  | (0.73,1.36) | (0.85,1.16) | (0.83,1.20) | (0.84,1.17) | (0.83,1.20) | (0.77,1.29) |
|  |  | M3 | 1.01±0.14 | 1.07±0.07 | 1.06±0.08 | 1.03±0.07 | 1.06±0.08 | 1.05±0.11 |
|  |  |  | (0.76,1.30) | (0.93,1.22) | (0.90,1.22) | (0.89,1.17) | (0.90,1.22) | (0.85,1.28) |
|  |  | M4 | 1.02±0.16 | 1.000±0.07 | 1.00±0.08 | 1.01±0.08 | 1.00±0.08 | 1.01±0.12 |
|  |  |  | (0.75,1.35) | (0.87,1.15) | (0.86,1.17) | (0.87,1.17) | (0.86,1.17) | (0.82,1.25) |
