# Supplementary material for: Genetic Association Analysis Using Sibship Data: A Multilevel Model Approach
Source: PLoS One. 2012 Feb 1;7(2):e31134. doi: 10.1371/journal.pone.0031134 (PMC3270036; doi:10.1371/journal.pone.0031134)
Supplement: Table S3 — Measures of power (M1) and type I error (M2–M4) of scenarios 5–8. (DOC) [file pone.0031134.s004.doc]

**Supporting Information**

**Table S3. Measures of power (M1) and type I error (M2-M4) of scenarios 5-8.**

| Scenario | Marker | S-TDT | SDT | CLR | GEEe | GEEi  &rGEEi | rGEEe | MLM | rMLM |
| --- | --- | --- | --- | --- | --- | --- | --- | --- | --- |
| 5 | M1 | 0.847 | 0.858 | 0.910 | 0.362 | 0.950 | 0.932 | 0.815 | 0.918 |
| Hypothesized proportion of DSPs=1.0 | M2 | 0.052 | 0.055 | 0.043 | 0.061 | 0.050 | 0.044 | 0.012 | 0.038 |
|  | M3 | 0.038 | 0.042 | 0.039 | 0.051 | 0.041 | 0.041 | 0.011 | 0.032 |
|  | M4 | 0.048 | 0.049 | 0.050 | 0.044 | 0.042 | 0.049 | 0.005 | 0.034 |
| 6 | M1 | 0.814 | 0.812 | 0.869 | 0.712 | 0.949 | 0.923 | 0.881 | 0.917 |
| Hypothesized proportion of DSPs=0.9 | M2 | 0.065 | 0.062 | 0.054 | 0.047 | 0.052 | 0.056 | 0.017 | 0.047 |
|  | M3 | 0.037 | 0.039 | 0.039 | 0.079 | 0.057 | 0.039 | 0.020 | 0.035 |
|  | M4 | 0.043 | 0.054 | 0.045 | 0.056 | 0.054 | 0.042 | 0.016 | 0.034 |
| 7 | M1 | 0.780 | 0.790 | 0.838 | 0.871 | 0.978 | 0.938 | 0.937 | 0.941 |
| Hypothesized proportion of DSPs=0.8 | M2 | 0.051 | 0.048 | 0.040 | 0.054 | 0.052 | 0.045 | 0.021 | 0.037 |
|  | M3 | 0.051 | 0.051 | 0.048 | 0.088 | 0.070 | 0.054 | 0.029 | 0.042 |
|  | M4 | 0.045 | 0.045 | 0.040 | 0.043 | 0.053 | 0.049 | 0.012 | 0.037 |
| 8 | M1 | 0.693 | 0.693 | 0.763 | 0.916 | 0.962 | 0.927 | 0.936 | 0.926 |
| Hypothesized proportion of DSPs=0.7 | M2 | 0.047 | 0.047 | 0.034 | 0.053 | 0.050 | 0.041 | 0.028 | 0.034 |
|  | M3 | 0.040 | 0.045 | 0.041 | 0.102 | 0.086 | 0.053 | 0.047 | 0.053 |
|  | M4 | 0.044 | 0.044 | 0.044 | 0.044 | 0.038 | 0.049 | 0.014 | 0.034 |
