# Supplementary material for: Genetic Association Analysis Using Sibship Data: A Multilevel Model Approach
Source: PLoS One. 2012 Feb 1;7(2):e31134. doi: 10.1371/journal.pone.0031134 (PMC3270036; doi:10.1371/journal.pone.0031134)
Supplement: Table S4 — Measures of parameter estimates (average OR, empirical standard error and 95%CI) of scenarios 5–8. (DOC) [file pone.0031134.s005.doc]

**Supporting Information**

**TABLE S4. Measures of parameter estimates (average OR, empirical standard error and 95%CI) of scenarios 5-8**.

| Scenario | Marker | CLR | GEEe | GEEi  &rGEEi | rGEEe | MLM | rMLM |
| --- | --- | --- | --- | --- | --- | --- | --- |
| 5 | M1 | 1.51±0.19 | 1.05±0.03 | 1.26±0.08 | 1.24±0.08 | 1.26±0.08 | 1.41±0.14 |
| Hypothesized proportion of DSPs=1.0 |  | (1.19,1.95) | (0.99,1.12) | (1.11,1.43) | (1.10,1.41) | (1.11,1.43) | (1.18,1.72) |
|  | M2 | 1.01±0.13 | 1.00±0.04 | 1.00±0.08 | 1.00±0.07 | 1.00±0.08 | 1.01±0.12 |
|  |  | (0.77,1.28) | (0.93,1.07) | (0.86,1.16) | (0.86,1.15) | (0.86,1.16) | (0.79,1.26) |
|  | M3 | 1.00±0.12 | 1.01±0.03 | 1.01±0.07 | 1.00±0.06 | 1.01±0.07 | 1.01±0.10 |
|  |  | (0.80,1.24) | (0.95,1.07) | (0.88,1.13) | (0.88,1.12) | (0.88,1.13) | (0.83,1.19) |
|  | M4 | 1.00±0.13 | 1.00±0.03 | 1.00±0.07 | 1.00±0.06 | 1.00±0.07 | 1.00±0.10 |
|  |  | (0.77,1.28) | (0.94,1.06) | (0.87,1.12) | (0.88,1.13) | (0.87,1.12) | (0.81,1.21) |
| 6 | M1 | 1.51±0.20 | 1.13±0.06 | 1.29±0.09 | 1.26±0.08 | 1.29±0.09 | 1.44±0.15 |
| Hypothesized proportion of DSPs=0.9 |  | (1.15,1.94) | (1.03,1.25) | (1.12,1.48) | (1.10,1.42) | (1.12,1.48) | (1.17,1.73) |
|  | M2 | 1.01±0.14 | 1.00±0.06 | 1.00±0.08 | 1.00±0.08 | 1.00±0.08 | 1.00±0.13 |
|  |  | (0.75,1.31) | (0.89,1.11) | (0.85,1.17) | (0.85,1.17) | (0.85,1.17) | (0.77,1.28) |
|  | M3 | 1.00±0.12 | 1.02±0.05 | 1.02±0.07 | 1.01±0.07 | 1.02±0.07 | 1.01±0.10 |
|  |  | (0.78,1.26) | (0.93,1.13) | (0.88,1.16) | (0.88,1.14) | (0.88,1.16) | (0.83,1.22) |
|  | M4 | 1.02±0.13 | 1.00±0.05 | 1.01±0.07 | 1.01±0.07 | 1.01±0.07 | 1.01±0.11 |
|  |  | (0.78,1.30) | (0.91,1.11) | (0.86,1.16) | (0.88,1.14) | (0.86,1.16) | (0.82,1.23) |
| 7 | M1 | 1.53±0.22 | 1.21±0.07 | 1.33±0.10 | 1.28±0.09 | 1.33±0.10 | 1.48±0.16 |
| Hypothesized proportion of DSPs=0.8 |  | (1.15,1.99) | (1.07,1.36) | (1.16,1.53) | (1.12,1.46) | (1.16,1.53) | (1.20,1.82) |
|  | M2 | 1.01±0.15 | 1.00±0.07 | 1.01±0.09 | 1.01±0.08 | 1.01±0.09 | 1.01±0.13 |
|  |  | (0.75,1.33) | (0.88,1.15) | (0.85,1.19) | (0.86,1.17) | (0.85,1.19) | (0.78,1.29) |
|  | M3 | 1.01±0.13 | 1.04±0.06 | 1.03±0.08 | 1.01±0.07 | 1.03±0.08 | 1.03±0.11 |
|  |  | (0.77,1.30) | (0.92,1.16) | (0.89,1.19) | (0.88,1.16) | (0.89,1.19) | (0.83,1.26) |
|  | M4 | 1.01±0.14 | 1.00±0.06 | 1.00±0.08 | 1.00±0.07 | 1.00±0.08 | 1.00±0.11 |
|  |  | (0.77,1.32) | (0.89,1.13) | (0.86,1.16) | (0.87,1.15) | (0.86,1.16) | (0.80,1.25) |
| 8 | M1 | 1.50±0.22 | 1.26±0.09 | 1.35±0.11 | 1.28±0.09 | 1.35±0.11 | 1.48±0.16 |
| Hypothesized proportion of DSPs=0.7 |  | (1.13,1.97) | (1.10,1.43) | (1.15,1.55) | (1.11,1.45) | (1.15,1.55) | (1.19,1.81) |
|  | M2 | 1.02±0.15 | 1.00±0.08 | 1.00±0.09 | 1.00±0.08 | 1.00±0.09 | 1.01±0.14 |
|  |  | (0.75,1.33) | (0.86,1.17) | (0.83,1.19) | (0.85,1.17) | (0.83,1.19) | (0.77,1.30) |
|  | M3 | 1.00±0.14 | 1.04±0.08 | 1.03±0.08 | 1.01±0.08 | 1.03±0.08 | 1.03±0.12 |
|  |  | (0.76,1.31) | (0.91,1.20) | (0.89,1.21) | (0.88,1.18) | (0.89,1.21) | (0.83,1.29) |
|  | M4 | 1.02±0.15 | 1.01±0.07 | 1.01±0.08 | 1.01±0.07 | 1.01±0.08 | 1.01±0.12 |
|  |  | (0.75,1.34) | (0.88,1.15) | (0.86,1.17) | (0.87,1.15) | (0.86,1.17) | (0.79,1.25) |
