# Supplementary material for: Genetic Association Analysis Using Sibship Data: A Multilevel Model Approach
Source: PLoS One. 2012 Feb 1;7(2):e31134. doi: 10.1371/journal.pone.0031134 (PMC3270036; doi:10.1371/journal.pone.0031134)
Supplement: Table S5 — Measures of power (M1), type I error (M2–M4) and parameter estimate (average OR, empirical standard error and 95%CI) of scenario 10. (DOC) [file pone.0031134.s006.doc]

**Supporting Information**

**Table S5. Measures of power (M1), type I error (M2-M4) and parameter estimate (average OR, empirical standard error and 95%CI) of scenario 10.**

|  | Marker | S-TDT | SDT | CLR | MLM | GEEe | GEEi & rGEEi | rGEEe | rMLM |
| --- | --- | --- | --- | --- | --- | --- | --- | --- | --- |
| Power and type I error rate | M1 | 0.857 | 0.863 | 0.917 | 0.825 | 0.080 | 0.942 | 0.936 | 0.924 |
|  | M2 | 0.052 | 0.046 | 0.054 | 0.014 | 0.069 | 0.050 | 0.050 | 0.040 |
|  | M3 | 0.052 | 0.059 | 0.049 | 0.015 | 0.070 | 0.047 | 0.048 | 0.040 |
|  | M4 | 0.041 | 0.039 | 0.047 | 0.013 | 0.050 | 0.055 | 0.056 | 0.044 |
| Parameter estimation | M1 | - | - | 1.51±0.18 | 1.26±0.08 | 1.06±0.12 | 1.26±0.08 | 1.24±0.08 | 1.42±0.14 |
|  |  | - | - | (1.18,1.89) | (1.11,1.42) | (0.85,1.32) | (1.11,1.42) | (1.09,1.40) | (1.16,1.71) |
|  | M2 | - | - | 1.00±0.13 | 1.00±0.08 | 1.00±0.12 | 1.00±0.08 | 1.00±0.07 | 1.00±0.11 |
|  |  | - | - | (0.76,1.28) | (0.85,1.15) | (0.77,1.24) | (0.85,1.15) | (0.85,1.15) | (0.79,1.23) |
|  | M3 | - | - | 1.01±0.12 | 1.01±0.06 | 1.02±0.11 | 1.01±0.06 | 1.01±0.06 | 1.01±0.10 |
|  |  | - | - | (0.80,1.27) | (0.89,1.15) | (0.83,1.25) | (0.89,1.15) | (0.89,1.13) | (0.84,1.22) |
|  | M4 | - | - | 1.01±0.12 | 1.00±0.07 | 1.01±0.11 | 1.00±0.07 | 1.00±0.06 | 1.00±0.10 |
|  |  | - | - | (0.79,1.26) | (0.88,1.14) | (0.83,1.25) | (0.88,1.14) | (0.88,1.14) | (0.81,1.23) |
